# Effects of elevated CO_2_ on photosynthetic traits of native and invasive C_3_ and C_4_ grasses

**DOI:** 10.1186/s12898-016-0082-z

**Published:** 2016-05-31

**Authors:** Heather A. Hager, Geraldine D. Ryan, Hajnal M. Kovacs, Jonathan A. Newman

**Affiliations:** School of Environmental Sciences, University of Guelph, Guelph, ON N1G 2W1 Canada; Department of Integrative Biology, University of Guelph, Guelph, ON N1G 2W1 Canada

**Keywords:** C_3_ photosynthesis, C_4_ photosynthesis, Climate change, Ecophysiology, Elevated CO_2_, Grasslands, Invasive species, Plant competition

## Abstract

**Background:**

Rising CO_2_ is expected to result in changes in plant traits that will increase plant productivity for some functional groups. Differential plant responses to elevated CO_2_ are likely to drive changes in competitive outcomes, with consequences for community structure and plant diversity. Many of the traits that are enhanced under elevated CO_2_ also confer competitive success to invasive species, and it is widely believed that invasive species will be more successful in high CO_2_. However, this is likely to depend on plant functional group, and evidence suggests that C_3_ plants tend to respond more strongly to CO_2_.

**Results:**

We tested the hypothesis that invasive species would be more productive than noninvasive species under elevated CO_2_ and that stronger responses would be seen in C_3_ than C_4_ plants. We examined responses of 15 grass species (eight C_3_, seven C_4_), classified as noninvasive or invasive, to three levels of CO_2_ (390, 700 and 1000 ppm) in a closed chamber experiment. Elevated CO_2_ decreased conductance and %N and increased shoot biomass and C/N ratio across all species. Differences between invasive and noninvasive species depended on photosynthetic mechanism, with more differences for traits of C_3_ than C_4_ plants. Differences in trait means between invasive and noninvasive species tended to be similar across CO_2_ levels for many of the measured responses. However, noninvasive C_3_ grasses were more responsive than invasive C_3_ grasses in increasing tiller number and root biomass with elevated CO_2_, whereas noninvasive C_4_ grasses were more responsive than invasive C_4_ grasses in increasing shoot and root biomass with elevated CO_2_. For C_3_ grasses, these differences could be disadvantageous for noninvasive species under light competition, whereas for C_4_ grasses, noninvasive species may become better competitors with invasive species under increasing CO_2_.

**Conclusions:**

The ecophysiological mechanisms underlying invasion success of C_3_ and C_4_ grasses may differ. However, given that the direction of trait differences between invasive and noninvasive grasses remained consistent under ambient and elevated CO_2_, our results provide evidence that increases in CO_2_ are unlikely to change dramatically the competitive hierarchy of grasses in these functional groups.

**Electronic supplementary material:**

The online version of this article (doi:10.1186/s12898-016-0082-z) contains supplementary material, which is available to authorized users.

## Background

Rising atmospheric CO_2_ is known to alter an array of plant traits, often resulting in enhanced plant growth. Elevated CO_2_ has been shown to enhance photosynthetic output, above- and below ground biomass production, and the concentration of photosynthate, resulting in higher C/N ratios [1, 2]. Water use efficiency, as a result of stomatal closure in high CO_2_, has also been shown to increase [3–5], contributing to increases in plant biomass through improved drought tolerance. Such changes can enhance primary productivity in a variety of grassland ecosystems, including shortgrass steppe [6], arid grasslands [7], calcareous grasslands [8], and tallgrass prairies [9].

Increased CO_2_ concentration can alter plant competition in cases where species respond differentially to changes [10]. Competitive outcomes are likely to be altered in favour of species responding positively to elevated CO_2_, with consequences for plant community composition and diversity. For example, global change factors, including elevated CO_2_, will likely alter the effects of invasive plants on native and managed ecosystems [11]. Invasive species may be more productive under elevated CO_2_ for several reasons. The success of invasive species is often greatest in novel, resource-rich environments, and ecosystem invasibility is also related to resource availability [11]. Also, many of the traits that are enhanced in high CO_2_ are also those that confer a competitive advantage to successful invaders [12]. Several important invasive species have been shown to respond positively to rising CO_2_. For example, the biomass of *Pueraria lobata* (kudzu) increased by 51 % in response to elevated CO_2_ [13]. Canada thistle (*Cirsium arvense*), widely considered to be one of the most invasive species in the continental United States, showed a 180 % increase in biomass under elevated CO_2_ [14]. In an even more extreme example, *Centaurea solstitialis* (yellow starthistle), one of California’s worst weeds, grew 600 % larger in elevated CO_2_ relative to ambient, while native plants responded much less strongly or not at all [15]. Within-species studies suggest that traits associated with invasion success, rather than just phylogenetic differences, may account for the response of invasive species to CO_2_. For example, Mozdzer and Megonigal [16] examined the responses of two different populations of the same grass species to elevated CO_2_ (North American-native and Eurasian-introduced genotypes of *Phragmites australis*) and found that the introduced genotype had stronger responses to CO_2_ for all ecophysiological traits measured.

Plant responses to elevated CO_2_ are highly dependent on plant functional group (e.g., photosynthetic mechanism, nitrogen fixation, reproductive system, growth form; [2]). Robinson et al.’s [2] meta-analysis of 152 plant species found the largest and most consistent differences between C_3_ and C_4_ plant groups. Plants with a C_4_ photosynthetic mechanism are adapted for low CO_2_ environments and contain a biochemical pump that concentrates CO_2_ at the site of carboxylation, thus reducing carbon loss through photorespiration. At current levels of CO_2_, the carboxylation function of Rubisco in C_4_ plants is thought to be near saturation. C_3_ plants do not possess this CO_2_ concentrating ability, and carbon gains are expected under elevated CO_2_ as the concentration gradient of CO_2_ from the air to the site of carboxylation increases. Of 365 C_3_ plant responses and 37 C_4_ plant responses to elevated CO_2_ measured, on average, plant biomass was significantly increased in C_3_ species but was unchanged in C_4_ species [2]. Additionally, the variance associated with C_4_ responses was substantially higher than for C_3_ plants [2], and this variability is reflected in the literature. For example, Ziska and Bunce [17] found that four of ten C_4_ species had higher biomass under elevated CO_2_, while eight of ten species had increased rates of photosynthesis, suggesting that not all C_4_ species are unresponsive. Additionally, a meta-analysis of C_3_ and C_4_ responses restricted to the Poaceae found that while C_3_ plant biomass increased by 44 % in response to elevated CO_2_, C_4_ biomass increased by 33 %, suggesting that responses are not readily predicted by photosynthetic mechanism alone [18].

Differences in the average growth responses of individual C_3_ and C_4_ plants have generally resulted in the predicted competitive outcomes when grown in mixtures. A meta-analysis of competition outcomes for different plant functional groups grown in elevated CO_2_ found that when grown in competition, C_3_ plants tended to outperform C_4_ plants [10]. However, this occurred only in high-nutrient conditions; there were no differences between these groups for low nutrient conditions, and nitrogen-fixing plant species tended to dominate over other plant groups [10]. Thus, functional groups such as C_3_ and N-fixing plants that have the ability to exploit enhanced resource availability under elevated CO_2_ are likely to be more competitive. Invasive species that fall into these categories are likely to become more aggressive invaders, potentially with increased success of C_3_ trees, shrubs, forbs, and grasses invading C_4_ grasslands. On the other hand, native and crop C_3_ plants may have a competitive advantage over potential invaders (e.g. invasion of C_4_ weeds in C_3_ crop fields; [19]). However, there is still much to be learned about C_4_ plant responses to elevated CO_2_, and exceptions to these general responses have been noted. For example, Owensby et al. [9] found that CO_2_ increased the production of C_4_ grasses but not C_3_ grasses in a three-year study of grassland ecosystems using open-top chambers.

Here, we test the hypothesis that plant invasive potential under elevated CO_2_ is dependent on photosynthetic mechanism using multiple species in a closed-chamber experiment. We examine responses to elevated CO_2_ in 15 grass species (eight C_3_ and seven C_4_) classified as either “noninvasive” or “invasive” (Table 1) and measured at two separate time points to account for possible CO_2_ acclimation phenomena. Specifically, we examine whether photosynthetic and morphological traits associated with productivity, competitive ability, and invasiveness are differentially altered in these groups under elevated CO_2_.

**Table 1:** List of plant species and their photosynthetic, invasiveness, and phylogenetic characteristics

Species name	Common name	Invasiveness	Subfamily (tribe)^a^	Seed source^b^
C_3_ photosynthesis				
*Brachypodium sylvaticum*	Slender false brome	Invasive	Pooideae	Collected: Grey County
*Bromus inermis*	Smooth brome	Invasive	Pooideae	Collected: Wellington County
*Dactylis glomerata*	Orchard grass	Invasive	Pooideae	Collected: Wellington County
*Elymus repens*	Quackgrass	Invasive	Pooideae	Collected: Wellington County
*Phalaris arundinacea*	Reed canary grass	Invasive	Pooideae	Collected: Wellington County
*Schedonorus arundinaceus* cv. KY-31 E-	Tall fescue	Invasive	Pooideae	T. Phillips, University of Kentucky
*Elymus virginicus*	Virginia wild rye	Noninvasive	Pooideae	Wildflower Farms, Ontario
*Lolium perenne* cv. Nui (A8385)	Perennial ryegrass	Noninvasive	Pooideae	D. Hume, AgResearch, New Zealand
C_4_ photosynthesis				
*Miscanthus sinensis*	Miscanthus	Invasive	Panicoideae (Paniceae)^c^	Jelitto Perennial Seed, Schwarmstedt, Germany
*Miscanthus giganteus*	Miscanthus	Invasive	Panicoideae (Paniceae)^c^	Mendel Biotechnology, Hayward, California
*Panicum miliaceum*	Proso millet	Invasive	Panicoideae (Paniceae)^d^	Collected: Wellington County
*Andropogon gerardii*	Big bluestem	Noninvasive	Panicoideae (Andropogoneae)^c^	Wildflower Farms, Ontario
*Bouteloua curtipendula*	Sideoats gramma	Noninvasive	Chloridoideae^d^	Wildflower Farms, Ontario
*Panicum virgatum* cv. Cave-in-Rock	Switchgrass	Noninvasive	Panicoideae (Paniceae)^d^	Ernst Conservation Seeds, Meadville, Pennsylvania
*Schizachyrium scoparium*	Little bluestem	Noninvasive	Panicoideae (Andropogoneae)^c^	Wildflower Farms, Ontario

^a^Based on GPWG II [46]

^b^Collected from field populations in southern Ontario, unless otherwise indicated

^c^NADP-me C_4_ photosynthetic subtype [20]

^d^NAD-me C_4_ photosynthetic subtype [20]

## Results

### Photosynthetic characteristics

There was a significant effect of time on photosynthetic response (Table 2) whereby photosynthesis was higher at 7 than 14 weeks of growth. However, this was dependent on plant species (time × species interaction; Table 2, Additional file 1a). Five species showed large decreases in photosynthetic rate at 14 weeks (C_3_: *Elymus virginicus*, C_4_: *Bouteloua curtipendula*, *Miscanthus giganteus*, *Miscanthus sinensis*, and *Panicum virgatum*), whereas only the C_3_*Schedonorus arundinaceus* showed a small increase, although none of the within-species changes were significant in a post hoc Tukey’s test. Pre-planned contrasts found no differences in photosynthetic rates between C_3_ and C_4_ plants or invasive and noninvasive plants at any CO_2_ levels at 14 weeks of growth (Table 3). Although not statistically significant, photosynthetic rate was 18.4 % higher in C_3_ than C_4_ plants at ambient CO_2_ (390 ppm), but differed by 0–1.7 % at elevated CO_2_ (not shown).

**Table 2:** Summary of ANOVA results

Source	Photo.	Cond.	SD (top)	SD (bot.)	SLA	Tillers	% N	% C	C:N	Shoot	Root
CO_2_		***					*		†	†	
	F_2,4_ = 0.5	*F*_*2,4*_ *=* *234.9*	F_2,4_ = 0.3	F_2,4_ = 0.3	F_2,4_ = 0.6	F_2,4_ = 0.5	*F*_*2,4*_ *=* *9.1*	F_2,4_ = 0.3	F_2,4_ = 6.3	F_2,4_ = 5.6	F_2,4_ = 4.3
SP	†	***	***	***	***	***	***	***	***	***	***
	F_12,24_ = 1.9	*F*_*12,24*_ *=* *16.5*	*F*_*12,24*_ *=* *41.3*	*F*_*12,24*_ *=* *98.0*	*F*_*12,24*_ *=* *10.8*	*F*_*12,24*_ *=* *44.7*	*F*_*14,28*_ *=* *14.4*	*F*_*14,28*_ *=* *9.5*	*F*_*14,28*_ *=* *13.1*	*F*_*14,26*_ *=* *11.4*	*F*_*14,26*_ *=* *21.2*
CO_2_ × SP									†		
	F_24,48_ = 1.1	F_24,48_ = 1.0	F_24,48_ = 0.8	F_24,48_ = 1.2	F_24,48_ = 1.3	F_24,43_ = 1.3	F_28,56_ = 1.5	F_28,56_ = 1.0	F_28,56_ = 1.6	F_28,52_ = 1.2	F_28,52_ = 1.0
T	**	*		†	*	**					
	*F*_*1,2*_ *=* *109.1*	*F*_*1,2*_ *=* *37.2*	F_1,2_ = 1.5	F_1,2_ = 10.8	*F*_*1,2*_ *=* *38.3*	*F*_*1,2*_ *=* *197.7*					
CO_2_ × T	F_2,4_ = 1.8	F_2,4_ = 0.2	F_2,4_ = 2.4	F_2,4_ = 0.1	F_2,4_ = 3.9	F_2,4_ = 1.1					
SP x T	†	*	*		***	***					
	F_12,24_ = 1.9	*F*_12,24_ = *2.3*	*F*_*12,24*_ *=* *2.8*	F_12,24_ = 1.7	*F*_*12,24*_ *=* *6.2*	*F*_*12,24*_ *=* *15.2*					
CO_2_ × SP × T			†	*	*	†					
	F_24,48_ = 1.1	F_24,48_ = 1.3	F_24,48_ = 1.7	*F*_*24,48*_ *=* *176*	*F*_*24,48*_ *=* *1.8*	F_24,41_ = 1.6					

Values in italics represent significant effects

^†^ P < 0.10; * P < 0.05; ** P < 0.01; *** P < 0.001

*Blank fields* not analysed, *SP* species, *T* time, *Photo*. photosynthetic rate, *Cond.* conductance, *SLA* specific leaf area, *SD* stomatal density, *bot.* bottom

**Table 3:** Results of pre-planned contrasts for photosynthetic and growth measures of C_3_ vs. C_4_ and invasive vs. noninvasive species groups at 14 weeks of growth (see Table 1)

	C_3_ vs. C_4_ photosynthesis			
	All species	Within CO_2_		
		390 ppm	700 ppm	1000 ppm
Photo.	F_1,44_ = 0.5	F_1,138_ = 1.6	F_1,138_ = 0.0	F_1,138_ = 0.0
Cond.	***	***	**	**
	*F*_*1,47*_ *=* *31.9*	*F*_*1,140*_ *=* *15.6*	*F*_*1,140*_ *=* *7.8*	*F*_*1,140*_ *=* *10.5*
SD (top)	***	**	***	***
	*F*_*1,45*_ *=* *12.3*	*F*_*1,112*_ *=* *10.0*	*F*_*1,112*_ *=* *16.4*	*F*_*1,112*_ *=* *15.0*
SD (bot.)	***	***	***	***
	*F*_*1,48*_ *=* *494.2*	*F*_*1,129*_ *=* *234.0*	*F*_*1,129*_ *=* *240.1*	*F*_*1,129*_ *=* *244.3*
SLA	*			
	*F*_*1,46*_ *=* *5.4*	F_1,140_ = 2.4	F_1,140_ = 0.6	F_1,140_ = 2.4
Tillers	***	***	***	***
	*F*_*1,37*_ *=* *319.5*	*F*_*1,109*_ *=* *165.5*	*F*_*1,107*_ *=* *90.0*	*F*_*1,107*_ *=* *121.1*
%N	***	***	***	***
	*F*_*1,28*_ *=* *131.2*	*F*_*1,84*_ *=* *58.7*	*F*_*1,84*_ *=* *57.2*	*F*_*1,84*_ *=* *28.0*
%C	***	*	**	
	*F*_*1,28*_ *=* *16.13*	*F*_*1,84*_ *=* *6.7*	*F*_*1,84*_ *=* *11.0*	*F*_1,84_ *=* 2.1
C:N	***	***	***	***
	*F*_*1,28*_ *=* *121.0*	*F*_*1,83*_ *=* *56.6*	*F*_*1,83*_ *=* *58.8*	*F*_*1,83*_ *=* *27.2*
Shoot	***	***	***	***
	*F*_*1,26*_ *=* *67.5*	*F*_*1,75*_ *=* *45.9*	*F*_*1,76*_ *=* *24.2*	*F*_*1,76*_ *=* *27.7*
Root	***	***	***	***
	*F*_*1,26*_ *=* *171.2*	*F*_*1,82*_ *=* *73.4*	*F*_*1,82*_ *=* *39.3*	*F*_*1,82*_ *=* *75.0*

	Invasive vs. Noninvasive											
	All species	C_3_ species	C_4_ species	Within CO_2_			C_3_ species within CO_2_			C_4_ species within CO_2_		
				390 ppm	700 ppm	1000 ppm	390 ppm	700 ppm	1000 ppm	390 ppm	700 ppm	1000 ppm
								†				
Photo.	F_1,44_ = 0.4	F_1,44_ = 0.2	F_1,44_ = 0.1	F_1,138_ = 0.4	F_1,138_ = 2.7	F_1,138_ = 0.0	F_1,138_ = 1.0	F_1,138_ = 3.0	F_1,138_ = 0.0	F_1,138_ = 0.2	F_1,138_ = 0.5	F_1,138_ = 0.1
				†				**				
Cond.	F_1,47_ = 1.9	F_1,47_ = 0.0	F_1,47_ = 0.0	F_1,140_ = 3.5	F_1,140_ = 0.2	F_1,140_ = 0.9	F_1,140_ = 1.9	*F*_*1,140*_ *=* *2.6*	F_1,140_ = 0.1	F_1,140_ = 0.0	F_1,140_ = 0.0	F_1,140_ = 0.0
	***		***	†	*	***				*	*	**
SD (top)	*F*_*1,45*_ *=* *12.3*	F_1,45_ = 1.3	*F*_*1,45*_ *=* *17.5*	F_1,112_ = 3.5	*F*_*1,112*_ *=* *6.6*	*F*_*1,112*_ *=* *11.4*	F_1,112_ = 1.2	F_1,112_ = 0.2	F_1,112_ = 1.0	*F*_*1,112*_ *=* *6.2*	*F*_*1,112*_ *=* *6.7*	*F*_*1,112*_ *=* *17.9*
		***	**				***	**	***	*	†	**
SD (bot.)	F_1,48_ = 0.1	*F*_*1,48*_ *=* *24.2*	*F*_*1,48*_ *=* *11.9*	F_1,129_ = 0.3	F_1,129_ = 0.4	F_1,129_ = 0.8	*F*_*1,129*_ *=* *13.7*	*F*_*1,129*_ *=* *8.2*	*F*_*1,129*_ *=* *13.8*	*F*_*1,129*_ *=* *5.9*	F_1,129_ = 3.0	*F*_*1,129*_ *=* *9.4*
	***	***		*		**	***		***			
SLA	*F*_*1,46*_ *=* *12.4*	*F*_*1,46*_ *=* *21.8*	F_1,46_ = 0.0	*F*_*1,140*_ *=* *6.2*	F_1,140_ = 0.1	*F*_*1,140*_ *=* *9.9*	*F*_*1,140*_ *=* *14.3*	F_1,140_ = 0.2	*F*_*1,140*_ *=* *12.5*	F_1,140_ = 0.2	F_1,140_ = 0.1	F_1,140_ = 0.5
	*	***	***		**		*	***	***		***	†
Tillers	*F*_*1,37*_ *=* *4.3*	*F*_*1,36*_ *=* *33.0*	*F*_*1,37*_ *=* *13.2*	F_1,110_ = 0.2	*F*_*1,107*_ *=* *8.1*	F_1,107_ = 2.3	*F*_*1,107*_ *=* *6.4*	*F*_*1,107*_ *=* *13.5*	*F*_*1,107*_ *=* *20.5*	F_1,111_ = 1.8	*F*_*1,107*_ *=* *14.3*	F_1,107_ = 2.8
		***					*	*	**			
% N	F_1,28_ = 0.7	*F*_*1,28*_ *=* *19.5*	F_1,28_ = 0.1	F_1,84_ = 1.7	F_1,84_ = 0.8	F_1,84_ = 1.1	*F*_*1,84*_ *=* *5.9*	*F*_*1,84*_ *=* *4.3*	*F*_*1,84*_ *=* *11.6*	F_1,84_ = 0.3	F_1,84_ = 0.1	F_1,84_ = 0.7
	†	***				*	*	*	**			
% C	F_1,28_ = 4.1	*F*_*1,28*_ *=* *20.4*	F_1,28_ = 0.4	F_1,84_ = 0.8	F_1,84_ = 0.0	*F*_*1,84*_ *=* *5.9*	*F*_*1,84*_ *=* *6.6*	*F*_*1,84*_ *=* *5.0*	*F*_*1,84*_ *=* *8.0*	F_1,84_ = 0.0	F_1,84_ = 0.1	F_1,84_ = 2.4
		***					*	*	***			
C:N	F_1,28_ = 0.1	*F*_*1,28*_ *=* *20.6*	F_1,28_ = 0.2	F_1,83_ = 1.3	F_1,83_ = 0.7	F_1,83_ = 1.8	*F*_*1,83*_ *=* *6.7*	*F*_*1,83*_ *=* *5.0*	*F*_*1,83*_ *=* *12.9*	F_1,83_ = 0.3	F_1,83_ = 0.1	F_1,83_ = 1.1
	***	†	**	***	**	***				*	†	**
Shoot	*F*_*1,26*_ *=* *39.7*	F_1,26_ = 3.0	*F*_*1,27*_ *=* *12.9*	*F*_*1,75*_ *=* *24.0*	*F*_*1,76*_ *=* *10.7*	*F*_*1,76*_ *=* *23.4*	F_1,75_ = 2.0	F_1,76_ = 0.4	F_1,75_ = 2.2	*F*_*1,75*_ *=* *6.5*	F_1,75_ = 3.2	*F*_*1,77*_ *=* *9.1*
	***	**	*	***	*	***	**		†			*
Root	*F*_*1,26*_ *=* *62.4*	*F*_*1,26*_ *=* *9.5*	*F*_*1,27*_ *=* *5.4*	*F*_*1,82*_ *=* *33.3*	*F*_*1,82*_ *=* *6.8*	*F*_*1,82*_ *=* *33.9*	*F*_*1,82*_ *=* *8.4*	F_1,82_ = 0.5	F_1,82_ = 3.9	F_1,83_ = 2.5	F_1,82_ = 0.0	*F*_*1,83*_ *=* *5.6*

Values in italics represent significant effects

^†^ P < 0.10; * P < 0.05; ** P < 0.01; *** P < 0.001

*Photo.* photosynthetic rate, *Cond*. conductance, *SD* stomatal density; *bot.* bottom, *SLA* specific leaf area

**Fig. 1:**
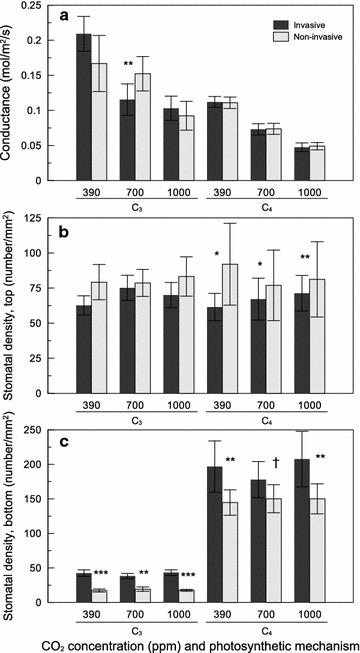
The effect of CO_2_ concentration (ppm), photosynthetic mechanism, and invasive status on **a** conductance, **b** adaxial stomatal density, and **c** abaxial stomatal density (mean ± SE). *Asterisks* represent the degree of significance between invasive status: ^†^P < 0.10, *P < 0.05, **P < 0.01, ***P < 0.001

There was a significant effect of CO_2_ on plant conductance, with lower conductance at higher CO_2_ concentrations (Fig. 1a; Table 2). Significant species, time, and species x time effects (Table 2) indicated that conductance was generally lower at 14 than 7 weeks, with the exception of *Schizachyrium scoparium*, which showed the opposite pattern. Contrasts at 14 weeks showed that conductance was higher in C_3_ than C_4_ plants, and this relationship held across all CO_2_ concentrations (Fig. 1a; Table 3). Invasive and noninvasive species had no detectable differences in conductance, with the exception of lower conductance in invasive than noninvasive C_3_ species at 700 ppm (Fig. 1a; Table 3).

Stomatal density differed among species, and these differences were dependent on time for the upper leaf surface and on time and CO_2_ concentration for the lower leaf surface (species × time and CO_2_ × species × time interactions, respectively; Table 2). For the upper surface, there was little change in stomatal density between 7 and 14 weeks except for *Andropogon gerardii,* which showed a large decrease. For the lower surface, stomatal density was generally greater at 14 than 7 weeks, but this pattern differed inconsistently for some species at some CO_2_ concentrations. Contrasts at 14 weeks showed that upper leaf stomatal density was lower overall in C_3_ than C_4_ plants, but this was inconsistent across CO_2_ levels, being higher in C_3_ than C_4_ plants at 700 ppm (Table 3). Upper stomatal density was lower overall in invasive than noninvasive species, and this pattern was driven by differences between invasive and noninvasive C_4_ species, with no differences between invasive and noninvasive C_3_ species (Fig. 1b; Table 3). However, absolute differences in upper leaf stomatal density were small. Lower leaf stomatal density was consistently lower in C_3_ than C_4_ plants across all CO_2_ levels, and was consistently higher in invasive than noninvasive C_3_ and C_4_ species across CO_2_ levels (Fig. 1c; Table 3).

**Fig. 2:**
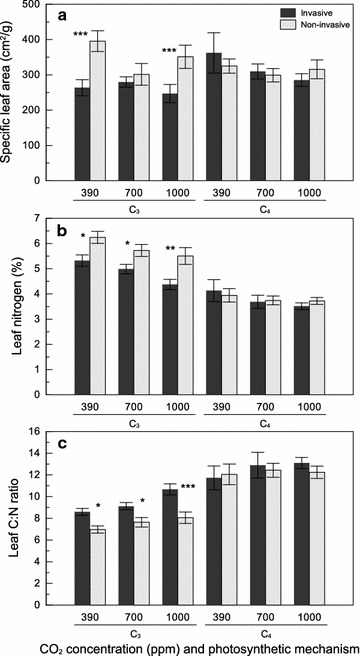
The effect of CO_2_ concentration (ppm), photosynthetic mechanism, and invasive status on **a** specific leaf area, **b** leaf N, and **c** leaf C:N ratio (mean ± SE). *Asterisks* represent the degree of significance between invasive status: ^†^P < 0.10, *P < 0.05, **P < 0.01, ***P < 0.001

Specific leaf area (SLA, unit leaf area per unit leaf weight) differed among species and with time, and those differences depended on CO_2_ concentration (Table 2). SLA decreased between 7 and 14 weeks for six of the species, and increased or showed no change over time for the remainder, with no clear trends among CO_2_ concentrations. Contrasts at 14 weeks showed lower overall SLA in C_3_ than C_4_ plants, but this pattern was not detected when CO_2_ levels were examined individually (Table 3). SLA was also lower in invasive than noninvasive C_3_ species, except at 700 ppm (Fig. 2a; Table 3).

### Nitrogen and carbon

Nitrogen concentration (%N) decreased significantly under elevated CO_2_ (Fig. 2b; Table 2). There was also an effect of species on %N, with highest concentrations in the C_3_ species *Lolium perenne, Elymus virginicus*, and *Phalaris arundinacea,* and lowest concentrations in the C_4_ species *Miscanthus sinensis, Miscanthus giganteus,* and *Bouteloua curtipendula* (Additional file 1b). Contrasts showed that %N was significantly higher for C_3_ than C_4_ plants at all CO_2_ concentrations (Table 3). %N was lower in invasive than noninvasive C_3_ species across CO_2_ levels but did not differ for C_4_ species (Fig. 2b; Table 3).

Carbon concentration (%C) differed among plant species (Table 2) and was lower in *Schedonorus arundinaceus* and *Lolium perenne* than in all other species. Contrasts revealed that  %C was slightly lower in C_3_ than C_4_ plants except at the highest CO_2_ level.  %C was higher in invasive than noninvasive C_3_ species across CO_2_ levels but did not differ for C_4_ species (Table 3).

There was an effect of species on the C/N ratio (Table 2), with highest C/N in the C_4_ species *Miscanthus sinensis, Miscanthus giganteus,Bouteloua curtipendula,* and *Andropogon gerardii,* and lowest C/N in the C_3_ species *Bromus inermis, Phalaris arundinacea, Elymus virginicus*, and *Lolium perenne.* Both CO_2_ and CO_2_ x species were weakly significant (Table 2), with C/N tending to increase under elevated CO_2_, but more for some species than others. Contrasts showed that differences in C/N followed a similar pattern to  %C. That is, C/N was lower in C_3_ than C_4_ plants across CO_2_ levels, and was higher in invasive than noninvasive C_3_ species across CO_2_ levels but did not differ for invasive and noninvasive C_4_ species (Fig. 2c; Table 3).

### Plant growth and dry mass

**Fig. 3:**
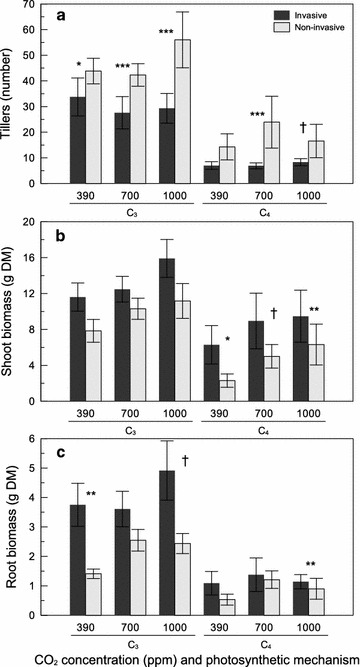
The effect of CO_2_ concentration (ppm), photosynthetic mechanism, and invasive status on **a** tillers, **b** shoot biomass, and **c** root biomass (mean ± SE). *DM* dry mass. *Asterisks* represent the degree of significance between invasive status: ^†^P < 0.10, *P < 0.05, **P < 0.01, ***P < 0.001

Tiller production was affected by species, time, and their interaction, but not CO_2_ (Table 2). Tiller number increased between 7 and 14 weeks for all species except *Andropogon gerardii,* which did not change. Contrasts at 14 weeks showed that tiller number was higher in C_3_ than C_4_ plants across CO_2_ levels (Table 3). Invasive C_3_ and C_4_ species had fewer tillers than their respective invasive species across all CO_2_ levels except for C_4_ plants at ambient CO_2_ (Fig. 3a; Table 3).

There was a significant effect of species on both shoot and root biomass (Table 2). Shoot biomass was significantly greater in *Elymus repens, Dactylis glomerata, Lolium perenne, Phalaris arundinacea, Schedonorus arundinaceus,* and *Panicum miliaceum* than in *Miscanthus sinensis,Bouteloua curtipendula,* and *Andropogon gerardii.* Root biomass was significantly greater in *Elymus repens, Dactylis glomerata, Lolium perenne, Phalaris arundinacea, Schedonorus arundinaceus, Bromus inermis, Elymus virginicus,* and *Brachypodium sylvaticum* than in *Miscanthus sinensis, Bouteloua curtipendula,* and *Panicum virgatum.* There was a weak effect of CO_2_ on shoot biomass whereby mass tended to increase under elevated CO_2_ (Table 2). Contrasts detected greater shoot and root mass in invasive than noninvasive species when pooled as well as separated by photosynthetic mechanism (Table 3). However, patterns were weaker when examined across CO_2_ levels. For C_4_ plants, invasive species had greater shoot mass across CO_2_ levels (Fig. 3b) and greater root mass at 1000 ppm (Fig. 3c). For C_3_ plants, invasive species had greater root mass at ambient and 1000 ppm, but no differences were detected in shoot mass across CO_2_ levels (Fig. 3b, c; Table 3).

## Discussion

### C_3_ vs. C_4_ responses to CO_2_

Elevated CO_2_ resulted in the typically expected changes [2, 18] for some photosynthetic and growth responses at 14 weeks in the grasses studied but not for others. As expected, conductance was greater for C_3_ than C_4_ grasses at all CO_2_ levels and decreased with increasing CO_2_. Similarly, %N was higher in C_3_ than C_4_ grasses at all CO_2_ levels and decreased with increasing CO_2_, whereas the opposite pattern held for C:N, most strongly due to the contribution of %N (however, Taylor et al. [20] raise the possibility that the commonly observed C_3_–C_4_ differences in grass leaf N could be a partial effect of phylogeny, which was not examined here). In contrast, photosynthetic rates are expected to be lower in C_3_ than C_4_ grasses at ambient CO_2_ and to increase more for C_3_ than C_4_ grasses with elevated CO_2_ (but see [18]). However, we detected no differences in photosynthetic rates between C_3_ and C_4_ grasses at 14 weeks. Although this result might have been caused by greenhouse conditions that were more optimal for C_3_ than C_4_ growth (but see [18]), such an effect should emphasize a greater increase in C_3_ than C_4_ photosynthetic rates with increases in CO_2_, which was not the case. Overall, photosynthetic rates decreased with time, and additional contrasts at 7 weeks detected the expected lower photosynthetic rates in C_3_ than C_4_ grasses at ambient CO_2,_ and a loss of that difference with elevated CO_2_ (Additional file 2). Decreasing photosynthetic rates over time could be attributed to increasing light limitation (although natural-light day-length had increased) and/or a CO_2_ acclimation response, for example, due to root restriction [21], with corresponding downregulation of photosynthetic enzymes [22, 23]. Indeed, photosynthetic rate decreased with time for more C_4_ than C_3_ grasses (4 of 7 vs. 1 of 8, respectively), but there was no change with time for the remaining C_4_ and 6 of the 7 remaining C_3_ grasses, so evidence for either mechanism of decline is equivocal.

Typical expected photosynthetic differences should also translate to biomass responses, with greater increases in productivity for C_3_ than C_4_ plants with elevated CO_2_ [10, 22]. We detected marginally significant increases in shoot biomass with increases in CO_2_, but the lack of CO_2_ x species interaction suggests that the increases were similar for C_3_ and C_4_ grasses. The lack of a root biomass or tiller number response to elevated CO_2_ corresponds with results for photosynthetic rate. The overall higher productivity of C_3_ than C_4_ grasses could be a result of potentially preferential conditions for C_3_ growth; i.e., C_4_ usually prefer high light and warmer, drier conditions than do C_3_ plants [22].

Finally, the responses of both SLA and stomatal density to elevated CO_2_ have been observed to vary inconsistently among grass species, even within photosynthetic mechanism. Although SLA is generally expected to decrease with increasing CO_2_ (e.g., [10, 18, 24]), studies of C_3_ grasses find that different species respond differently to elevated CO_2_ [24–26]. Our results were consistent with previous findings in that the effect of CO_2_ varied among species. Overall, however, SLA was lower for C_3_ than C_4_ grasses, indicating that C_3_ grasses tended to have thicker or denser leaf tissue. Although stomatal density has been proposed to decrease with elevated CO_2_ because of energetic costs [27] or redistribution of stomata due to increases in vascular tissue [28], stomatal density has been found to differ by species in response to elevated CO_2_, even within photosynthetic mechanism [28] and genus (e.g., *Panicum* [29]). Species-specific differences would explain our nonsignificant CO_2_ effect but significant CO_2_ x species x time interaction. Although we were unable to detect CO_2_-based differences within species (within the species x CO_2_ x time interaction), trends indicate different responses to elevated CO_2_ for within-genus pairs (i.e., *Elymus, Miscanthus,* and *Panicum*). The lack of a strong CO_2_ main effect on stomatal density suggests that differences in conductance among CO_2_ levels are a result of physiological control of stomatal aperture behaviour, rather than plasticity in stomatal density [28].

### Invasive vs. noninvasive responses to CO_2_

Although we detected differences between invasive and noninvasive grasses for some photosynthetic and growth responses across CO_2_ levels, the differences frequently depended on the photosynthetic mechanism. Invasive C_3_ grasses had lower SLA and leaf N content, and higher leaf C and C:N ratio than did noninvasive C_3_ grasses, whereas invasive C_4_ grasses had lower upper leaf stomatal density than noninvasive C_4_ grasses. When the responses did not differ by photosynthetic mechanism, they were always in the same direction. That is, invasive grasses had higher stomatal density on the lower leaf surface, produced fewer tillers, and had greater shoot and root biomass than native grasses for both C_3_ and C_4_ grasses.

Differences between invasive and noninvasive grasses were consistent across CO_2_ levels for many of the traits measured (i.e., magnitudes of the differences were <10 %). Thus, invasive and noninvasive C_3_ grasses responded similarly to elevated CO_2_ for lower leaf stomatal density, SLA, leaf N, and C:N. Invasive and noninvasive C_4_ grasses responded similarly to elevated CO_2_ for lower leaf stomatal density and number of tillers. In contrast, invasive grasses were either more or less responsive than noninvasive grasses to elevated CO_2_ for some traits.

For C_3_ plants, noninvasive grasses responded to elevated CO_2_ with increases in tiller numbers, whereas invasive grasses did not, as well as with greater per-gram increases in root biomass than did invasive grasses (although absolute increases were similar). Thus, under the nonlimiting nutrient and water conditions of our experiment, noninvasive C_3_ grasses appear to invest more in belowground tissue and clonal expansion under elevated CO_2_ than do invasive C_3_ grasses, which could be disadvantageous in competition for light. However, we did not measure plant height or total leaf area, which would allow better determination of this potential trade-off.

For C_4_ plants, the difference between invasive and noninvasive upper leaf stomatal density decreased with elevated CO_2_, but persisted. Noninvasive grasses also had greater per-gram increases in shoot and root biomass than did invasive grasses (slightly greater absolute increases). Thus, although the invasive grasses always had greater absolute shoot and root biomass than the noninvasive grasses, noninvasive C_4_ grasses may become less disadvantaged in competition with invasive C_4_ grasses under elevated CO_2_. This idea contrasts with previous findings of potentially increased success of invasive grasses under elevated CO_2_ [30, 31].

Given that the direction of differences between invasive and noninvasive grasses did not change with elevated CO_2_ for any of the measured traits, we conclude that elevated CO_2_ is unlikely to alter significantly the competitive hierarchy of species within these functional groups given that many of these traits are considered indicative of invasive ability [32, 33]. Our findings echo those of previous studies that found no effects of elevated CO_2_ on the relative growth rate rankings of 19 species [34] or on the competitive rankings of 14 species pairs [31] from multiple functional groups, suggesting that “winners always win” [34]. However, chamber and field experiments examining competitive outcomes under elevated CO_2_ as well as in combination with various resource limitations (e.g., [35]) will be required to determine which species are winners under other conditions because individual plant responses to CO_2_ may not scale predictably to the community level [10, 36].

### Invasive traits of grasses

Overall differences between the invasive and noninvasive grasses were not always in the expected directions based on previous large-scale multispecies trait analyses (e.g., [37–39]. For example, we found that invasive grasses had lower SLA and leaf N than noninvasive grasses, although their photosynthetic rates were similar. However, the invasive grasses we studied had greater biomass allocation to shoot and root production than the noninvasive grasses, indicating higher nitrogen productivity [40]. The greater shoot biomass but lower tiller production of invasive grasses suggests that they were taller or had greater total leaf area than the noninvasive grasses, and they may have had an early higher growth rate advantage. In a greenhouse experiment, Reichmann et al. [41] also found that an invasive grass was able to maintain greater biomass than three native grasses, even though its initially higher SLA and relative growth rate converged with those of the natives over time. A field study that surveyed one invasive and three noninvasive C_4_ grasses also found that the invasive grass had lower SLA and leaf N but higher photosynthetic activity, suggesting higher nitrogen productivity, and the invasive grass began its growing season earlier than the natives [42]. Thus, invasive grasses may be successful because of early season advantages that allow competitive resource pre-emption [41], and further research should pursue this area of inquiry. We note also that quantitative syntheses lumping functional groups, experimental environments, and different physiological traits into trait groups may be obscuring some trait relations that could be important determinants of invasive success in certain species groups.

Overall, invasive species had fewer stomata on the top leaf surface than did noninvasive species, although this relationship was driven by the C_4_ grasses and was not statistically significant in the C_3_ grasses. To our knowledge, stomatal density has not been examined previously as a potential trait related to invasion success. However, in an extensive quantitative review of stomatal distribution, Muir [43] concluded that the proportion of stomata on each leaf surface is highly constrained by selective pressures to maximize photosynthesis rates while minimizing fitness costs. Minimizing the number of stomata on the upper leaf surface could reduce the risk of infection by foliar pathogens [43]. Thus, it is possible that some invasive plants are escaping natural enemies via altered stomatal distribution. This idea remains to be tested.

## Conclusion

Our experimental design allowed us to examine traits in a suite of species for different plant functional groups over time. Plant traits associated with increased invasion success are not always enhanced in invasive species under elevated CO_2_, and the ecophysiological mechanisms underlying invasion success of C_3_ and C_4_ grasses may differ. Given that the direction of trait differences between invasive and noninvasive grasses remained consistent under ambient and elevated CO_2_, our results provide evidence that increases in CO_2_ are unlikely to change dramatically the competitive hierarchy of grasses in these functional groups. A more complete model of invasive species responses to global change will require knowledge of how ecophysiological responses are likely to be mediated by factors such as light, nutrients, and herbivory.

## Methods

### CO_2_ growth chambers

The experiment was conducted in the E.C. Bovey Greenhouse at the University of Guelph, Ontario, in nine CO_2_-controlled plexiglass closed-top chambers arranged in a 3 × 3 square. Chambers were constructed and operated according to Grodzinski et al. [44]; they were 82 (height) × 52 × 45 cm and were computer controlled to maintain CO_2_, temperature (23 °C), and humidity (~40 %) levels using an Argus Greenhouse Control System (Argus, Surrey, British Columbia). We used three CO_2_ concentrations that are within the range of the projected increase by the year 2100 [45]: ambient (390 ppm) and two elevated (700 and 1000 ppm). The nine chambers were blocked according to a light gradient in the greenhouse, with one chamber of each CO_2_ concentration per block, for a total of 3 blocks. Lighting followed a 16:8 light/dark cycle. Supplementary artificial metal halide lights (approx. 150 μmol/m^2^/s in the absence of daylight) were used when natural light fell below 600 μmol/m^2^/s. Maximum external ambient light levels during the experimental period ranged from 2120 μmol/m^2^/s (October) to 1371 μmol/m^2^/s (December; estimated interior max. of 1000–1570 μmol/m^2^/s); these were 25–65 % of external light levels in August (max. 3032 μmol/m^2^/s).

### Plant material

Fifteen grass species (eight C_3_ and seven C_4_ species; see Table 1 for details and sources) were chosen for the experiment based on invasive status and seed availability. These species grow and can co-occur in pastures, grasslands, and roadside ditches, and *Miscanthus giganteus* is currently cultivated as a bioenergy feedstock, in Ontario and elsewhere in North America. Species were classified as invasive or noninvasive based on information from several databases: the Invasive Species Compendium (http://www.cabi.org/isc/); Ontario Ministry of Agriculture and Food *Ontario Weeds* (http://www.omafra.gov.on.ca/english/crops/facts/ontweeds/weedgal.htm), and Urban Forest Associates Inc. (http://ufora.ca/index.php/resources/invasive-species/). Many of these species are well-known invaders.

Grasses were germinated from seed at their CO_2_ treatment concentrations in greenhouse flats with LC-1 potting soil (Sun Gro-sunshine soil mix containing Canadian Sphagnum peat moss, coarse perlite, organic starter nutrient charge, Gypsum and dolomitic limestone). Three weeks after planting, seedlings were transferred into PVC pots (0.6 cm thick, 7.6 cm diameter PVC pipe cut to 45.7 cm height [1.73 L] and the bottom covered with mesh for drainage) containing the same potting mix. Each species was replicated once per chamber and three times per CO_2_ concentration for a total of 189 pots. Plants were watered ad libitum with alternating deionized and fertilized water (1.25 g/L N-P-K, 20-8-20). On days when photosynthesis was measured, all chambers received deionized water on the morning of data collection. Plants were grown for 14 weeks; any inflorescences that grew during this time were removed, dried, and weighed. At the end of the experiment, plants were harvested and separated into shoots and roots. Although root growth was extensive, roots were not observed to fill the pot volume. Roots were thoroughly washed, and all material was dried for at least 48 h at 55 °C in a forced air oven before being weighed.

### Measurement of plant traits

We measured photosynthetic rate, conductance, vegetative tiller number, and stomatal density at two time points over the course of the experiment (~7 and 14 weeks post-germination). Photosynthesis and conductance were measured using a portable infrared gas analyzer (LI-6400 Portable Photosynthesis System; LI-COR, Lincoln, Nebraska). The 2 × 3 cm LI-COR leaf clamp had an opaque LED light source (LI-6400-02B red/blue LED #670) set to 1600 μmol/m^2^/s and a CO_2_ injector (LI-6400-01 CO_2_ Injector System) that controlled the clamp chamber concentration to that of the growth chamber in which each plant was grown. The fully expanded, upper canopy leaf was measured between 9 am and 4 pm on data collection days. Due to time and daylight constraints, measurements were staggered such that plants from different blocks were measured on different days. After clamping the leaf into the LI-COR, each plant was allowed to acclimate to the light intensity until readings stabilized. An automatic logger was then initiated to record values every 20 s for 2 min (total of six measurements per species), which were subsequently averaged. Most of the leaf blades were not wide enough to cover the entire 2 × 3 cm leaf clamp. In these cases, the leaf was marked while still in the clamp, removed from the plant, and the width at each end measured using callipers; area was calculated as the area of a trapezoid. The leaf segment was then dried for 48 h at 55 °C in a forced air oven and used to calculate specific leaf area (SLA; leaf area to dry mass ratio). This tissue was then analyzed for carbon and nitrogen content (second time point only) using an elemental analyzer (vario Max CN analyzer, Elementar Analysesysteme Gmbh, Hanau, Germany).

A small section of leaf blade directly adjacent to the clamp section was used for taking cuticle prints from both the top and bottom of the leaf blade. A thin film of clear nail polish was brushed onto the cuticle. Once dried, the polish was removed with clear tape and placed onto a microscope slide. The total number of stomata was counted under a light microscope at 40× magnification and expressed as the number per area (in mm^2^) of plant tissue on both the top and bottom prints.

### Statistical analysis

Responses that were measured only at 14 weeks were analysed using a blocked split-plot design with CO_2_ as the whole-plot factor and species as a sub-plot factor, where individual chambers constituted the unit of replication. Responses that were measured at 7 and 14 weeks were analysed using the same design with an additional split-plot effect of time to account for the repeated measures. All analyses were performed using mixed effects ANOVA with species, CO_2_, and time as fixed factors, and block as a random factor. All block-factor interactions (except the highest order interaction) were included as error terms. Box-Cox transformation was used to homogenize the residual variance, and examination of the residuals following transformation suggested that assumptions of ANOVA were met. Two species (*Brachypodium sylvaticum* and *Phalaris arundinacea*) were excluded from analyses of photosynthesis, conductance, and stomatal density due to missing values. For each response variable at 14 weeks, we conducted several pre-planned contrasts: C_3_ vs. C_4_, invasive vs. noninvasive, C_3_ invasive vs. C_3_ noninvasive, C_4_ invasive vs. C_4_ noninvasive, and all interactions involving CO_2_. Analyses were conducted in JMP 10.0 and 12.0 (SAS Institute, Cary, NC). In text and figures, we report untransformed means and standard errors as a measure of data dispersion. Individual plant species means and standard errors are provided in Additional file 1 in the supplemental material for all CO_2_ concentrations and time points.

## Additional files

10.1186/s12898-016-0082-z (a) Mean ± SE for individual species across CO_2_ concentrations for responses measured at 7 and 14 weeks, (b) mean ± SE for individual species across CO_2_ concentrations for responses measured at 14 weeks only.
10.1186/s12898-016-0082-z Results of contrasts for photosynthetic rate at 7 weeks.

## Abbreviations

C: carbon
C_3_: Calvin cycle photosynthetic pathway
C_4_: Hatch-Slack cycle photosynthetic pathway
CO_2_: carbon dioxide
N: nitrogen
ppm: parts per million
SLA: specific leaf area
ANOVA: analysis of variance

## References

[1] Stiling P, Cornelissen T (2007). How does elevated carbon dioxide (CO_2_) affect plant–herbivore interactions? A field experiment and meta-analysis of CO_2_-mediated changes on plant chemistry and herbivore performance. Global Change Biol.

[2] Robinson EA, Ryan GD, Newman JA (2012). A meta-analytical review of the effects of elevated CO_2_ on plant-arthropod interactions highlights the importance of interacting environmental and biological variables. New Phytol.

[3] Drake BG, Gonzàlez-Meler MA, Long SP (1996). More efficient plants: a consequence of rising atmospheric CO_2_?. Annu Rev Plant Physiol Plant Mol Biol.

[4] Kirkham MB, He H, Bolger TP, Lawlor DJ, Kanemasu ET (1991). Leaf photosynthesis and water use of big bluestem under elevated carbon dioxide. Crop Sci.

[5] Nie D, He H, Mo G, Kirkham MB, Kanemasu ET (1992). Canopy photosynthesis and evapotranspiration of rangeland plants under doubled carbon dioxide in closed-top chambers. Agricult Forest Meteorol.

[6] Morgan JA, Lecain DR, Mosier AR, Milchunas DG (2001). Elevated CO_2_ enhances water relations and productivity and affects gas exchange in C_3_ and C_4_ grasses of the Colorado shortgrass steppe. Global Change Biol.

[7] Smith SD, Huxman TE, Zitzer SF, Charlet TN, Housman DC, Coleman JS (2000). Elevated CO_2_ increases productivity and invasive species success in an arid ecosystem. Nature.

[8] Stöcklin J, Leadley PW, Körner C (1997). Community and species level responses to elevated CO_2_ in designed calcareous grassland species. Acta Oecol.

[9] Owensby CE, Coyne PI, Ham JM, Auen LM, Knapp AK (1993). Biomass production in a tallgrass prairie ecosystem exposed to ambient and elevated CO_2_. Ecol Appl.

[10] Poorter H, Navas M (2003). Plant growth and competition at elevated CO_2_: on winners, losers and functional groups. New Phytol.

[11] Bradley BA, Blumenthal DM, Wilcove DS, Ziska LH (2009). Predicting plant invasions in an era of global change. Trends Ecol Evol.

[12] Dukes JS, Mooney HA (1999). Does global change increase the success of biological invaders?. Trends Ecol Evol.

[13] Sasek TW, Strain BR (1988). Effects of carbon dioxide enrichment on the growth and morphology of kudzu (*Pueraria lobata*). Weed Sci.

[14] Ziska LH (2003). Evaluation of the growth response of six invasive species to past, present and future atmospheric carbon dioxide. J Exp Bot.

[15] Dukes JS, Chiariello NR, Loarie SR, Field CB (2011). Strong response of an invasive plant species (*Centaurea solstitialis* L.) to global environmental changes. Ecol Appl.

[16] Mozdzer TJ, Megonigal JP (2012). Jack-and-master trait responses to elevated CO_2_ and N: a comparison of native and introduced *Phragmites australis*. PLoS ONE.

[17] Ziska LH, Bunce JA (1997). Influence of increasing carbon dioxide concentration on the photosynthetic and growth stimulation of selected C_4_ crops and weeds. Photosynth Res.

[18] Wand SJE, Midgley GF, Jones MH, Curtis PS (1999). Responses of wild C_2_ and C_3_ grass (Poaceae) species to elevated atmospheric CO_2_ concentration: a meta-analytic test of current theories and perceptions. Global Change Biol.

[19] Dukes JS, Mooney HA, Hobbs RJ (2000). Will the increasing atmospheric CO_2_ concentration affect the success of invasive species?. Invasive Species in a Changing World.

[20] Taylor SH, Hulme SP, Rees M, Ripley BS, Woodward FI, Osborne CP (2010). Ecophysiological traits in C_3_ and C_4_ grasses: a phylogenetically controlled screening experiment. New Phytol.

[21] Arp WJ (1991). Effects of source-sink relations on photosynthetic acclimation to elevated CO_2_. Plant Cell Environ.

[22] Sage RF, Kubien DS (2003). *Quo vadis* C_4_? An ecophysiological perspective on global change and the future of C_4_ plants. Photosyn Res.

[23] Leakey ADB, Ainsworth EA, Bernacchi CJ, Rogers A, Long SP, Ort DR (2009). Elevated CO_2_ effects on plant carbon, nitrogen, and water relations: six important lessons from FACE. J Exp Bot.

[24] Roumet C, Laurent G, Roy J (1999). Leaf structure and chemical composition as affected by elevated CO_2_: genotypic responses of two perennial grasses. New Phytol.

[25] Luo Y, Field CB, Mooney HA (1994). Predicting responses of photosynthesis and root fraction to elevated [CO_2_]_a_: interactions among carbon, nitrogen, and growth. Plant, Cell Environ.

[26] Roumet C, Bel MP, Sonie L, Jardon F, Roy J (1996). Growth response of grasses to elevated CO_2_: a physiological plurispecific analysis. New Phytol.

[27] Franks PJ, Beerling DJ (2009). Maximum leaf conductance driven by CO_2_ effects on stomatal size and density over geologic time. Proc Natl Acad Sci USA.

[28] Taylor SH, Franks PJ, Hulme SP, Spriggs E, Christin PA, Edwards EJ, Woodward FI, Osborne CP (2012). Photosynthetic pathway and ecological adaptation explain stomatal trait diversity amongst grasses. New Phytol.

[29] Tipping C, Murray DR (1999). Effects of elevated atmospheric CO_2_ concentration on leaf anatomy and morphology in *Panicum* species representing different photosynthetic modes. Int J Plant Sci.

[30] Nagel JM, Huxman TE, Griffin KL, Smith SD (2004). CO_2_ enrichment reduces the energetic cost of biomass construction in an invasive desert grass. Ecology.

[31] Manea A, Leishman MR (2011). Competitive interactions between native and invasive exotic plant species are altered under elevated carbon dioxide. Oecologia.

[32] Pysek P, Richardson DM, Nentwig W (2008). Traits associated with invasiveness in alien plants: Where do we stand?. Biological Invasions.

[33] Drenovsky RE, Grewell BJ, D’Antonio CM, Funk JL, James JJ, Molinari N, Parker IM, Richards CL (2012). A functional trait perspective on plant invasion. Ann Bot.

[34] Temme AA, Liu JC, Cornwell WK, Cornelissen JHC, Aerts R (2015). Winners always win: growth of a wide range of plant species from low to future high CO_2_. Ecol Evol.

[35] Wong SC, Osmond CB (1991). Elevated atmospheric partial pressure of CO_2_ and plant growth. III. Interactions between *Triticum aestivum* (C_3_) and *Echinochloa frumentacea* (C_4_) during growth in mixed culture under different CO_2_, N nutrition and irradiance treatments, with emphasis on below-ground responses estimated using the δ^13^ values of root biomass. Aus J Plant Physiol.

[36] Taylor K, Potvin C (1997). Understanding the long-term effect of CO_2_ enrichment on a pasture: the importance of disturbance. Can J Bot.

[37] Baruch Z, Goldstein G (1999). Leaf construction cost, nutrient concentration, and net CO_2_ assimilation of native and invasive species in Hawaii. Oecologia.

[38] Ordonez A, Wright IJ, Olff H (2010). Functional differences between native and alien species: a global-scale comparison. Funct Ecol.

[39] van Kleunen M, Weber E, Fischer M (2010). A meta-analysis of trait differences between invasive and non-invasive plant species. Ecol Lett.

[40] Lambers H, Chapin FS, Pons TL (1998). Plant physiological ecology.

[41] Reichmann LG, Schwinning S, Polley HW, Fay PA. Traits of an invasive grass conferring an early growth advantage over native grasses. J Plant Ecol. 2016. **(in press)**.

[42] González-Rodríguez AM, Baruch Z, Palomo D, Cruz-Trujillo G, Jiménez MS, Morales D (2010). Ecophysiology of the invader Pennisetum setaceum and three native grasses in the Canary Islands. Acta Oecol.

[43] Muir CD (2015). Making pore choices: repeated regime shifts in stomatal ratio. Proc R Soc B.

[44] Grodzinski B, Schmidt JM, Watts B, Taylor J, Bates S, Dixon MA (1999). Regulating plant/insect interactions using CO_2_ enrichment in model ecosystems. Adv Space Res.

[45] Ipcc A, Stocker TF, Qin D, Plattner GK, Tignor M, Allen SK, Boschung J, Nauels A, Xia Y, Bex V, Midgley PM (2013). Summary for policyamakers. Climate Change 2013: the physical science basis. Contribution of working group I to the fifth assessment report of the intergovernmental panel on climate change.

[46] Grass Phylogeny Working Group II (2012). New grass phylogeny resolves deep evolutionary relationships and discovers C_4_ origins. New Phytol.

[47] Hager HA, Ryan GD, Kovacs HM, Newman JA. (2015) Effects of elevated CO_2_ on photosynthetic traits of native and invasive C_3_ and C_4_ grasses 2012 to 2013 [South-central Ontario, Canada]. Agri-Environmental Research Data Repository, University of Guelph. http://www.hdl.handle.net/10864/TZBTY.

