# Supplementary material for: Effects of elevated CO2 on photosynthetic traits of native and invasive C3 and C4 grasses
Source: BMC Ecol. 2016 May 31;16:28. doi: 10.1186/s12898-016-0082-z (PMC4888642; doi:10.1186/s12898-016-0082-z)
Supplement: Supplementary file 2 — 10.1186/s12898-016-0082-z Results of contrasts for photosynthetic rate at 7 weeks. [file 12898_2016_82_MOESM2_ESM.pdf]

Additional File 2. Results of contrasts for photosynthetic rate at 7 weeks.

Hager et al. Effects of elevated CO_2 on photosynthetic traits of native and invasive C_3 and C_4 grasses.

Table S2. Results of contrasts for photosynthetic rate at 7 weeks compared to 14 weeks.

	C_3 vs. C_4			
	All species	within CO_2		
Photosynthesis		390 ppm	700 ppm	1000 ppm
7 weeks	** **$F_{1,44}$ = 10.1**	*** **$F_{1,138}$ = 26.0**	$F_{1,138}$ = 2.0	$F_{1,138}$ = 0.8
14 weeks	$F_{1,44}$ = 0.5	$F_{1,138}$ = 1.6	$F_{1,138}$ = 0.0	$F_{1,138}$ = 0.0

Values in bold represent significant effects. †P < 0.10; *P < 0.05; **P < 0.01; ***P < 0.001.

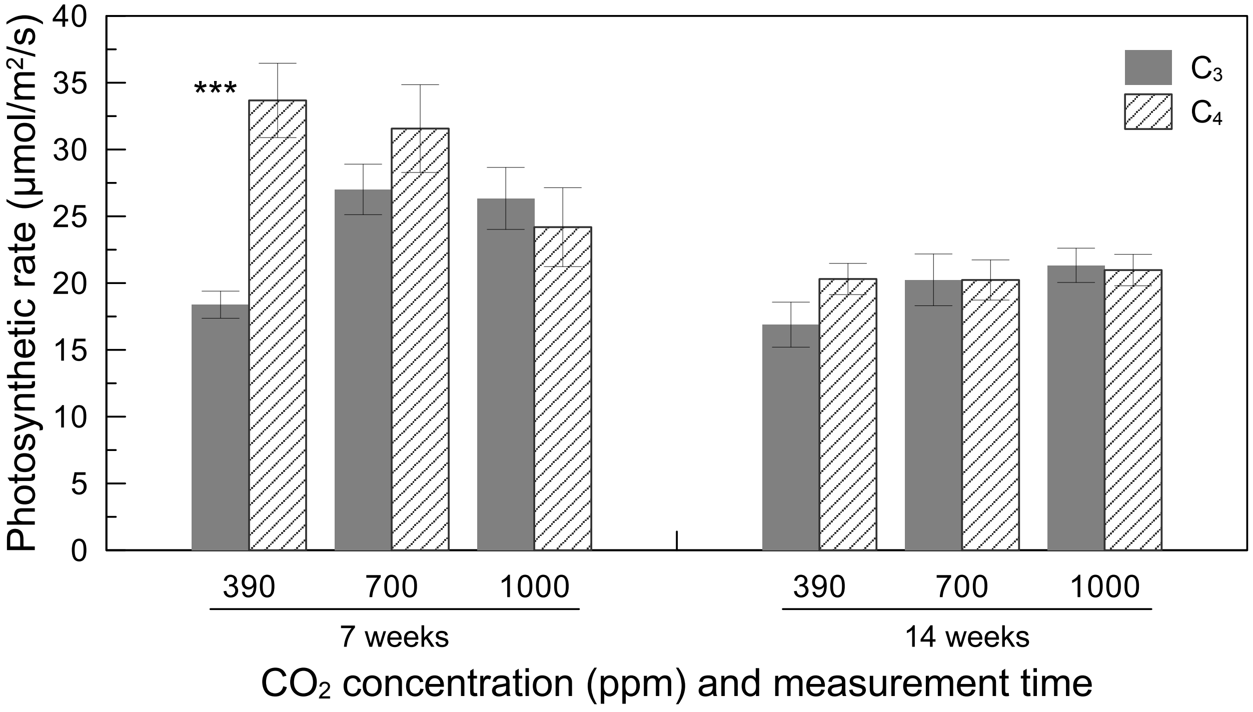

Figure S2. The effect of CO_2 concentration, measurement time, and photosynthetic mechanism on photosynthetic rate.
